# How Job Crafting Dimensions Differentially Moderate the Translation of Work Conditions into Stress Perceptions

**DOI:** 10.3390/bs15060793

**Published:** 2025-06-09

**Authors:** Giovanni Di Stefano, Elena Lo Piccolo, Lavinia Cicero

**Affiliations:** 1 Department of Psychology, Educational Science and Human Movement, University of Palermo, 90128 Palermo, Italy; 2 Department of Psychology “Renzo Canestrari”, Alma Mater Studiorum, University of Bologna, 40126 Bologna, Italy; elena.lopiccolo2@unibo.it; 3 Department of Theoretical and Applied Sciences, eCampus University, 22060 Novedrate, Italy; lavinia.cicero@uniecampus.it

**Keywords:** job crafting, work stress, effort–reward imbalance, job demands–resources, proactive behavior

## Abstract

Job crafting—employees’ proactive modification of their work—has gained attention as a potential stress management strategy. This study examined how job crafting dimensions moderate relationships between work conditions and stress perceptions. Integrating Effort–Reward Imbalance and Job Demands–Resources models, we tested whether three job crafting dimensions (increasing structural resources, social resources, and challenging demands) moderate the translation of factual work conditions into stress perceptions. Survey data from 376 Italian employees revealed that factual effort and reward indicators positively predicted their perceived counterparts. Contrary to expectations, increasing structural resources amplified rather than buffered the effort perception relationship, suggesting that certain crafting strategies may heighten rather than reduce awareness of work demands. As hypothesized, increasing social resources buffered the effort relationship and strengthened the reward relationship. Increasing challenging demands showed no significant moderating effects. These findings reveal that job crafting dimensions have differential rather than uniformly positive effects on stress perception processes. While social crafting appears consistently beneficial, structural crafting may have unintended consequences under certain conditions. Organizations should recognize that job crafting interventions require nuanced implementation. The study advances theory by demonstrating boundary conditions for job crafting effectiveness and challenging assumptions about its uniformly positive effects.

## 1. Introduction

The contemporary workplace is characterized by unprecedented levels of complexity and change, with employees facing escalating demands while simultaneously managing diminishing resources and uncertain career prospects. This evolving landscape has made work-related stress a critical concern for both individuals and organizations, with substantial implications for employee well-being, productivity, and organizational effectiveness (European Agency for Safety and Health at Work, 2022; European Foundation for the Improvement of Living and Working Conditions, 2024). Despite extensive research on workplace stress, a fundamental gap remains in our understanding of how employees can proactively modify their perception and management of stressors, particularly regarding the distinction between objective work conditions and their subjective appraisal.

The relationship between objective workplace characteristics and perceived stress represents a complex phenomenon that has long challenged organizational researchers. While environmental stressors exist independently of individual perception, the stress experience itself is fundamentally shaped by cognitive appraisal processes (Lazarus & Folkman, 1984). This dual nature of stress—as both an objective environmental condition and a subjective psychological experience—necessitates theoretical frameworks that can capture both aspects while explaining the mechanisms through which they interact. Recent developments in work stress theory have highlighted the need for integrated approaches that consider not only what stressors employees face but also how they actively shape their work environment to manage these stressors (Tims et al., 2022).

Job crafting, defined as the self-initiated changes employees make to optimize their job demands and resources (Gordon et al., 2018; Harju et al., 2021; Kardas, 2023; Petrou et al., 2018; Tims et al., 2012; Wrzesniewski & Dutton, 2001), has emerged as a promising mechanism through which individuals can influence their stress experience. However, the specific processes through which different job crafting dimensions affect the translation of objective stressors into perceived stress remain poorly understood. This gap is particularly evident when considering how job crafting might differentially moderate the relationships between objective and perceived aspects of both effort and reward in the workplace.

The present study addresses these gaps by integrating two complementary theoretical frameworks. The Effort–Reward Imbalance (ERI) model (Siegrist, 1996) provides specific content domains for understanding work stress and identifying effort–reward dynamics as critical determinants of employee well-being. The Job Demands–Resources (JD-R) model (Demerouti et al., 2001) offers a process framework for understanding how employees can proactively modify their work environment through resource-building strategies. By integrating these models, we examine how job crafting—conceptualized as a resource-building intervention rooted in JD-R theory—moderates the relationship between objective and perceived components of the ERI model.

This integration is theoretically significant for three reasons. First, it addresses calls in the literature for multi-level approaches to work stress that consider both environmental conditions and individual agency (Parker et al., 2019). Second, it responds to the need for a more nuanced understanding of job crafting effects, moving beyond simple main effects to examine how different crafting strategies may have differential impacts on stress processes (Zhang & Parker, 2019). Third, it contributes to the emerging literature on the potential “dark side” of proactive behaviors, recognizing that job crafting may not uniformly reduce stress but may instead have complex, sometimes paradoxical effects (Harju et al., 2021).

Therefore, the primary objective of this study is to examine how three dimensions of job crafting—increasing structural job resources, increasing social job resources, and increasing challenging job demands—differentially moderate the relationships between objective and perceived effort and reward. By doing so, we aim to contribute to both theoretical understanding and practical applications regarding how employees can effectively manage workplace stress through proactive job modification strategies.

## 2. Literature Review and Theoretical Framework

### 2.1. Distinguishing Factual Indicators from Subjective Perception

The distinction between factual workplace characteristics and their subjective evaluation represents a fundamental conceptual and methodological issue in occupational stress research. Transaction theory implies that stress is neither in the environmental input nor in the person but reflects the conjunction of a person with certain motives and beliefs with an environment whose characteristics pose harm, threats, or challenges depending on the person’s characteristics (Lazarus, 1990). This transactional perspective necessitates a careful examination of both environmental conditions and their psychological appraisal (Semmer et al., 2019).

The conceptualization of stress has evolved from simple stimulus-response models to complex transactional frameworks. Psychological stress results from the interplay of system variables and processes, depending on an appraisal by the person that the person–environment relationship at any given moment is one of harm, threat, or challenge (Lazarus, 1990). This fundamental insight suggests that while environmental conditions provide the context for stress, the stress experience itself emerges only after cognitive appraisal processes have occurred.

Building on established job analysis methodology (Harvey, 1991; Morgeson & Humphrey, 2006), we distinguish between factual job characteristics and their subjective evaluation. Our “standardized factual indicators” represent an attempt to capture verifiable aspects of the work environment through structured self-report. The critical distinction lies in what respondents are asked to report. Factual indicators query observable, verifiable characteristics: “Does your workday exceed 8 h?” (yes/no), “Are you required to perform multiple tasks simultaneously?” (yes/no). These items minimize interpretive variance by focusing on concrete, binary assessments of job features.

In contrast to factual indicators, perceived measures capture the evaluative and experiential aspects of work stress. Using validated scales from the ERI model (Siegrist, 1996), these measures employ Likert-type response formats that inherently invite subjective interpretation. Such items explicitly ask for personal evaluation of the stress experience rather than documentation of factual conditions.

The maintenance of both factual and perceptual levels of measurement serves several theoretical purposes. First, it allows examination of the appraisal process itself—how environmental conditions are transformed into stress experiences through cognitive mediation. Second, it addresses the fundamental question in stress research regarding the relative contribution of “objective” conditions versus subjective appraisal to health outcomes. By measuring both levels, we can examine not only direct effects but also the processes through which job crafting might modify the translation of factual conditions into perceived stress.

### 2.2. Integrating Content and Process: A Dual-Framework Approach to Work Stress

The integration of the Effort–Reward Imbalance (ERI) and Job Demands–Resources (JD-R) models represents a theoretically grounded approach that leverages the complementary strengths of each framework. This integration is not merely additive but synergistic, addressing limitations inherent in using either model in isolation while providing a more comprehensive understanding of work stress processes.

The ERI model (Siegrist, 1996) offers a content-specific framework that identifies critical stress-inducing conditions in the workplace. Its focus on the reciprocity principle—the expectation that efforts expended should be balanced by adequate rewards—captures a fundamental aspect of the employment relationship. The model’s strength lies in its specificity: it identifies concrete domains of effort (time pressure, interruptions, responsibility, overtime, physical demands) and reward (salary, esteem, career opportunities, job security) that consistently predict employee health outcomes across cultures and occupations (Siegrist & Li, 2016).

Conversely, the JD-R model (Bakker & Demerouti, 2007) provides a process framework that explains how workplace characteristics influence employee well-being through two distinct pathways: the health impairment process (high demands leading to strain) and the motivational process (resources fostering engagement). Critically, the JD-R model incorporates individual agency through concepts like job crafting, recognizing that employees are not passive recipients of job characteristics but active agents who can modify their work environment (Tims & Bakker, 2010).

Our integration addresses these complementary limitations through a dual-level approach. At the content level, we use the ERI model to specify which job characteristics are most relevant for work stress: effort expenditure and reward attainment. At the process level, we employ the JD-R framework to understand how job crafting operates as a resource-building strategy that can modify the relationship between these specific stressors and their perception.

This integration is theoretically justified by recent developments in the stress literature. Van Vegchel et al. (2005) noted that while ERI and JD-R models share common ground in recognizing the importance of job resources, they differ in their emphasis on specific mechanisms. Our approach builds on their observation by using ERI to identify what needs to be addressed (effort–reward imbalance) and JD-R to explain how it can be addressed (through job crafting).

By integrating the content specificity of the ERI model with the process orientation of the JD-R model, we develop a more complete theoretical framework that (1) identifies which aspects of work are most critical for stress (effort–reward imbalance), (2) explains how these objective conditions translate into subjective experiences, and (3) specifies how individual proactive behaviors (job crafting) can modify these relationships.

### 2.3. Job Crafting as Proactive Adaptation Behavior

Job crafting represents a specific form of proactive work behavior through which employees actively reshape their work experiences to achieve better alignment between job characteristics and personal needs, abilities, and preferences (Tims & Bakker, 2010; Wrzesniewski & Dutton, 2001). This conceptualization positions job crafting as distinct from reactive coping mechanisms, emphasizing its anticipatory and constructive nature.

Within our integrated ERI-JD-R framework, we conceptualize job crafting as a form of proactive adaptation behavior—distinct from reactive coping—that operates through bottom-up job redesign. Unlike coping, which represents responses to existing stressors, job crafting involves anticipatory modifications to prevent stress or enhance resources (Bindl & Parker, 2011). Unlike top-down job redesign, which is organizationally initiated, job crafting represents employee-initiated modifications to task, relational, and cognitive boundaries of work (Tims et al., 2012; Wrzesniewski & Dutton, 2001).

We adopt Tims et al.’s (Tims et al., 2012) conceptualization for three theoretically grounded reasons. First, their operationalization is explicitly embedded within the JD-R framework, making it ideally suited for examining how crafting behaviors modify demands and resources. Second, this approach captures observable behaviors rather than cognitive reframing, allowing for clearer measurement of actual workplace modifications. Third, meta-analytic evidence demonstrates that the behavioral dimensions identified by Tims et al. show consistent patterns of relationships with work outcomes (Rudolph et al., 2017).

Following Tims and Bakker (Tims & Bakker, 2010), job crafting comprises four behavioral dimensions:

1. Increasing Structural Job Resources: Behaviors aimed at enhancing autonomy, skill variety, and development opportunities;
2. Increasing Social Job Resources: Behaviors directed at building social support, seeking feedback, and enhancing supervisory coaching;
3. Increasing Challenging Job Demands: Proactively seeking new responsibilities and volunteering for challenging projects;
4. Decreasing Hindering Job Demands: Efforts to minimize emotionally, cognitively, or physically demanding aspects of work.

Within the stress process, job crafting serves as a proactive adaptation mechanism rather than reactive coping. This distinction is crucial: unlike coping, which responds to existing stressors, job crafting involves anticipatory modifications to prevent stress or enhance resources before problems arise. The primary goal is achieving better person–job fit through proactive adaptation.

### 2.4. Focus on Promotion-Oriented Job Crafting Dimensions

The decision to focus exclusively on the three “increasing” dimensions of job crafting while excluding the fourth dimension (decreasing hindering job demands) is grounded in both theoretical considerations and empirical evidence.

The fundamental distinction between the three increasing dimensions and the decreasing dimension lies in their underlying motivational orientations. The three increasing dimensions reflect promotion-focused behaviors oriented toward growth, development, and positive outcomes. In contrast, decreasing hindering job demands represents a prevention-focused strategy aimed at avoiding negative outcomes and minimizing threats (Lichtenthaler & Fischbach, 2019).

Meta-analytic evidence consistently demonstrates differential patterns. While the three increasing dimensions show positive relationships with work engagement (rc ranging from 0.35 to 0.45), the decreasing dimension often displays non-significant or even negative relationships with desirable outcomes (Rudolph et al., 2017). This pattern suggests that decreasing demands may represent withdrawal or disengagement rather than the proactive adaptation we aim to study. In other studies, decreasing hindering job demands showed a different pattern of correlation with peer-rated work engagement compared with the other three job crafting behaviors (Eguchi et al., 2016; Nielsen & Abildgaard, 2012). Lastly, the validated Italian version of the Job Crafting Scale (Cenciotti et al., 2016) demonstrated superior psychometric properties when focusing on the three increasing dimensions, with confirmatory factor analyses revealing better model fit indices for the three-factor solution.

Within our framework examining job crafting as proactive adaptation behavior, the three increasing dimensions align coherently with the constructive, resource-building nature of proactive behavior. By excluding the decreasing dimension, we maintain conceptual clarity and focus on job crafting behaviors that represent genuine attempts to optimize the work environment rather than withdraw from it.

### 2.5. Hypothesis Development

Based on our integrated theoretical framework, we develop hypotheses regarding how job crafting dimensions moderate the relationships between standardized factual indicators and perceived stress.

Following the ERI model and transactional stress theory, we expect that factual workplace characteristics will positively predict their perceived counterparts:

**H1a:**  *Standardized factual effort indicators will be positively related to perceived effort*.

**H1b:**  *Standardized factual reward indicators will be positively related to perceived reward*.

According to COR theory (Hobfoll, 1989, 2002), the acquisition of resources can offset the impact of demands. By increasing structural resources such as autonomy, workers may experience greater control over their tasks, improving person–environment fit and reducing the perceived impact of objective demands:

**H2a:**  *Increasing structural job resources moderates the positive relationship between factual effort indicators and perceived effort, such that the relationship will be weaker at higher levels of structural resource crafting*.

The literature on social support consistently demonstrates its buffering effect against stress (Cohen & Wills, 1985). When workers actively seek social resources, they receive both instrumental and emotional support, helping them reinterpret work demands as less threatening:

**H2b:**  *Increasing social job resources moderates the positive relationship between factual effort indicators and perceived effort, such that the relationship will be weaker at higher levels of social resource crafting*.

Challenge demands, unlike hindrance demands, can be positively perceived as they offer growth opportunities (Crawford et al., 2010). Workers who actively seek challenges might perceive objective demands as development opportunities rather than burdens:

**H2c:**  *Increasing challenging job demands moderates the positive relationship between factual effort indicators and perceived effort, such that the relationship will be weaker at higher levels of challenge crafting*.

Workers who proactively increase their structural resources might be more aware of and better appreciate objective rewards. Greater development opportunities and autonomy might lead workers to feel more valued by the organization:

**H3a:**  *Increasing structural job resources moderates the positive relationship between factual reward indicators and perceived reward, such that the relationship will be stronger at higher levels of structural resource crafting*.

Actively seeking social resources can enhance awareness of available rewards through social comparison and discussions with colleagues. Social support itself can serve as an additional reward that amplifies existing objective rewards:

**H3b:**  *Increasing social job resources moderates the positive relationship between factual reward indicators and perceived reward, such that the relationship will be stronger at higher levels of social resource crafting*.

Workers who actively seek challenges might perceive objective rewards as more meaningful, as they represent recognition of their engagement in stimulating tasks:

**H3c:**  *Increasing challenging job demands moderates the positive relationship between factual reward indicators and perceived reward, such that the relationship will be stronger at higher levels of challenge crafting*.

## 3. Materials and Methods

### 3.1. Participants and Procedure

This study employed a purposive convenience sampling strategy designed to capture a diverse range of Italian employees while maintaining methodological feasibility. We recruited participants through professional networks and organizational contacts, specifically targeting multiple organizations to enhance generalizability beyond single-organization studies.

We initially contacted Human Resources departments in 10 organizations across Southern Italy. Of these, five organizations agreed to participate, representing five major sectors: public administration (31% of final sample), healthcare (24%), education (18%), financial services (15%), and manufacturing (12%).

Prior to data collection, we obtained written consent from organizational management, with explicit agreements regarding voluntary participation, complete confidentiality, aggregate-only reporting, and no employer access to individual data. Employees were recruited through internal communications emphasizing the study’s academic nature and voluntary participation.

Data collection occurred in March 2025 through an online survey platform ensuring anonymity. The survey required approximately 20–25 min to complete. To minimize common method bias, we implemented several procedural remedies (Podsakoff et al., 2003): scale format variation, psychological separation through different instructional sets, and repeated anonymity assurances.

Of approximately 850 employees who received the invitation, 376 completed all required items (44.2% effective response rate). The mean age of the respondents was 46.86 years (SD = 7.70), and 67 percent of them were female; 9.6 completed junior high school education, 59.6 percent had a high school diploma, and 30.9 percent had some college education or above. Their average tenure in the current workplace was 15.89 years (SD = 7.76).

### 3.2. Measures

Standardized Factual Indicators of Effort and Reward. We developed standardized factual indicators to capture verifiable job characteristics that exist independently of individual evaluation and capture job features that could, in principle, be verified through external sources (e.g., work schedules, job descriptions, organizational policies). While collected via self-report for practical reasons, these items query factual rather than evaluative information, following established precedent in job analysis research where employee reports of factual job characteristics show high convergence with supervisor ratings and observational data (Dierdorff & Wilson, 2003; Harvey, 1991; Spector & Jex, 1998), using dichotomous response formats to minimize interpretive variance. The checklist was developed through (a) initial item generation based on ERI dimensions, (b) expert review by two organizational psychologists, (c) pilot testing with 20 respondents, (d) item refinement, and (e) final validation. We deliberately chose dichotomous (yes/no) response formats for three reasons: (1) Theoretical: To maximize distinction from subjective evaluation by asking for factual verification rather than degree of agreement (2) Methodological: Binary responses for factual items reduce response bias and enhance clarity (Dolnicar et al., 2011) (3) Practical: Pilot testing revealed that respondents found binary formats clearer for factual questions. The factual effort dimension consists of Business Indicators (A1–A3) (e.g., “My workday exceeds 8 h daily”) and Job Indicators (M1–M3) (e.g., “My job requires performing multiple tasks simultaneously”). The factual reward dimension comprises Career Indicators (C1–C3) (e.g., “I am aware of the career prospects for my position”) and Salary Indicators (R1–R3) (e.g., “Additions to regular salary are provided”), totaling 12 items with yes/no responses. We deliberately chose dichotomous formats to maximize distinction from subjective evaluation, reduce response bias, and enhance clarity based on pilot testing feedback.

Perceived Effort and Perceived Reward. The ERI questionnaire short version (Montano et al., 2016; Siegrist et al., 2009) was used. The Effort scale (3 items, α = 0.73) includes items like “I have constant time pressure due to a heavy workload.” The Reward scale (7 items, α = 0.76) includes items like “Considering all my efforts and achievements, I receive the respect and prestige I deserve at work.” Responses use 4-point scales (1 = completely disagree to 4 = completely agree).

Job crafting. Job crafting was measured with the Italian version of the Job Crafting Scale (Cenciotti et al., 2016). Following theoretical and empirical justification, we employed only three dimensions: increasing structural job resources (5 items, α = 0.69, e.g., “I try to develop my capabilities”), increasing social job resources (5 items, α = 0.79, e.g., “I ask my supervisor to coach me”), and increasing challenging job demands (5 items, α = 0.82, e.g., “When an interesting project comes along, I offer myself proactively”). Items use 7-point frequency scales (1 = never to 7 = always).

### 3.3. Analytical Strategy

Moderated hierarchical regression analyses were conducted using PROCESS macro for SPSS 26 and Process v4.2 (Hayes, 2022). Main effects were entered first, followed by interaction terms. All continuous predictors were mean-centered. We tested models both with and without demographic covariates (age, gender, education, tenure) to assess robustness.

## 4. Results

### 4.1. Preliminary Analyses: Demographic Effects

Before testing our hypotheses, we examined whether demographic factors influenced our key variables.

Significant gender differences emerged across multiple variables. Women reported lower standardized factual effort indicators (*M* = 2.48 vs. 3.19 for men, *t* = −6.28, *p* < 0.001) but higher factual reward indicators (*M* = 2.68 vs. 2.14, *t* = 5.46, *p* < 0.001). Women also reported higher perceived reward (*M* = 2.46 vs. 2.27, *t* = 3.22, *p* = 0.001). Regarding job crafting, men reported higher levels of increasing challenging demands (M = 5.20 vs. 4.81, *t* = 2.99, *p* = 0.003), while women showed marginally higher social job resources (M = 2.99 vs. 2.74, *t* = −1.98, *p* = 0.049).

Age showed significant correlations with several variables. Older employees reported lower factual effort indicators (*r* = −0.32, *p* < 0.001) but higher factual reward indicators (*r* = 0.15, *p* = 0.004) and perceived reward (*r* = 0.28, *p* < 0.001). Age was negatively correlated with increasing challenging demands (*r* = −0.12, *p* = 0.023), suggesting younger employees engage more in challenge-seeking behaviors.

Education level significantly affected multiple variables. University graduates reported higher factual effort (M = 3.29) compared to high school (M = 2.61) and junior high (M = 2.44) graduates (*F* = 10.26, *p* < 0.001). A similar pattern emerged for factual rewards (*F* = 20.27, *p* < 0.001). University graduates also showed higher levels of all three job crafting dimensions, particularly structural resources (*F* = 9.07, *p* < 0.001) and social resources (*F* = 9.97, *p* < 0.001).

Given these substantial demographic differences, we included gender, age, and education as covariates in all moderation analyses. Importantly, the inclusion of these covariates did not alter the pattern or significance of our hypothesized moderation effects, though some main effects were attenuated.

### 4.2. Descriptive Statistics and Correlations Among Variables

Table 1 presents means, standard deviations, and correlations among variables.

The positive correlations between factual indicators and their perceived counterparts support H1a (*r* = 0.329, *p* < 0.01) and H1b (*r* = 0.212, *p* < 0.01).

### 4.3. Hypothesis Testing

Table 2 and Table 3 present moderated regression results.

Contrary to H2a, the interaction between factual effort indicators and increasing structural resources was significant but in the opposite direction (*b* = 0.082, *p* < 0.05). Rather than attenuating the relationship, structural resource crafting amplified it. H2a was not supported. The negative main effect of factual effort (*b* = −0.292) reflects the relationship at the mean levels of the moderator, not the overall association (which remains positive as per the correlation in Table 1). Supporting H2b, increasing social resources significantly moderated the relationship (*b* = −0.046, *p* < 0.05), attenuating the effect of factual effort on perceived effort. H2c was not supported, as increasing challenging demands showed no significant moderating effect (*b* = 0.005, *p* = 0.865).

H3a was not supported (*b* = 0.016, *p* = 0.646). H3b was supported, with increasing social resources strengthening the reward relationship (*b* = 0.091, *p* < 0.001). H3c was not supported (*b* = −0.033, *p* = 0.111).

To probe the significant interactions, we conducted simple slope analyses and created interaction plots.

**Figure 1:**
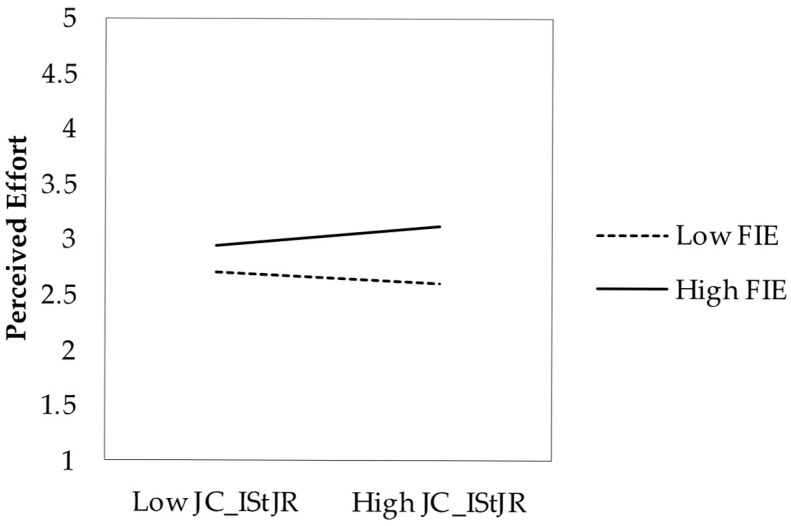
Moderating effect of increasing structural job resources on the relationship between factual indicators of effort and perceived effort. Note. FIE = factual indicators of effort; JC_IStJR = increasing structural job resources.

Figure 1 illustrates the moderating effect of increasing structural job resources on the relationship between standardized factual effort indicators and perceived effort. Simple slopes analysis revealed that the relationship between factual and perceived effort was stronger at high levels (+1 SD) of structural resource crafting (*b* = 0.243, SE = 0.044, *t* = 5.57, *p* < 0.001) compared to low levels (−1 SD) (*b* = 0.112, SE = 0.052, *t* = 2.17, *p* < 0.05). The Johnson–Neyman analysis indicated that for values of increasing structural job resources below 4.84 (approximately 16.3% of the sample), the relationship between factual and perceived effort was not statistically significant.

**Figure 2:**
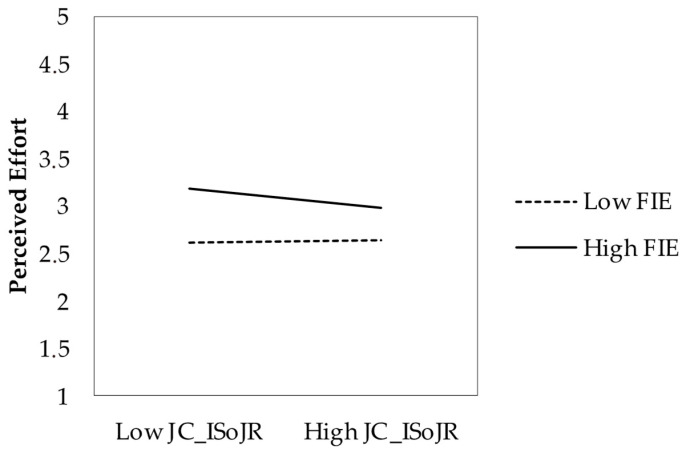
Moderating effect of increasing social job resources on the relationship between factual indicators of effort and perceived effort. Note. FIE = factual indicators of effort; JC_ISoJR = increasing social job resources.

Figure 2 depicts the moderating effect of increasing social job resources. The simple slopes analysis showed that the relationship between factual and perceived effort was stronger at low levels (−1 SD) of social resource crafting (*b* = 0.264, SE = 0.042, *t* = 6.24, *p* < 0.001) compared to high levels (+1 SD) (*b* = 0.156, SE = 0.039, *t* = 4.03, *p* < 0.001). The Johnson–Neyman analysis revealed that for values of increasing social job resources above 5.16 (approximately 4.3% of the sample), the relationship was no longer statistically significant.

**Figure 3:**
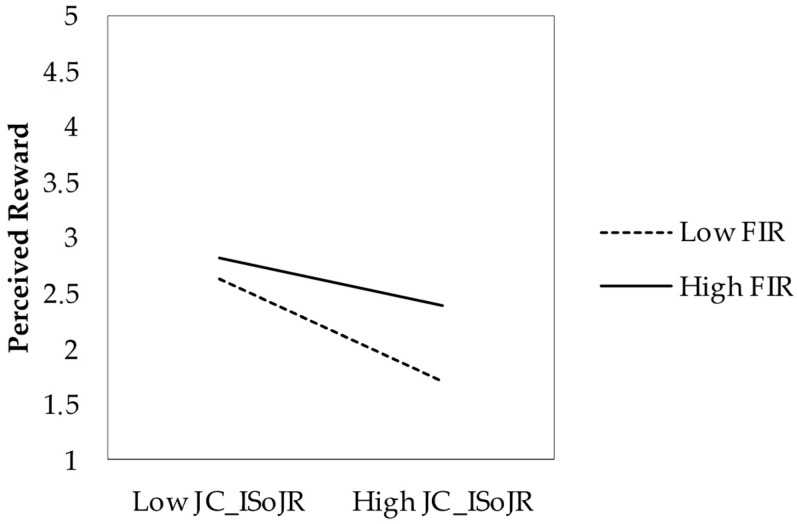
Moderating effect of increasing social job resources on the relationship between factual indicators of reward and perceived reward. Note. FIR = factual indicators of reward; JC_ISoJR = increasing social job resources.

Figure 3 shows the moderating effect of increasing social job resources on the reward relationship. Simple slopes analysis indicated that the relationship between factual and perceived reward was stronger at high levels (+1 SD) of social resource crafting (*b* = 0.245, SE = 0.037, *t* = 6.62, *p* < 0.001) compared to low levels (−1 SD) (*b* = 0.030, SE = 0.037, *t* = 0.81, *p* = 0.416, non-significant). The Johnson–Neyman analysis showed that for values below 2.08 (approximately 31.2% of the sample), the relationship between factual and perceived reward was not statistically significant.

## 5. Discussion

The results contradict our theoretical prediction based on COR theory and require careful interpretation. The positive interaction coefficient indicates that at higher levels of structural job resources crafting, the relationship between objective and perceived effort becomes stronger, not weaker, as we hypothesized. Our most theoretically provocative finding—that increasing structural job resources amplifies rather than attenuates the effort perception relationship—challenges fundamental assumptions in both JD-R and COR theories. This ‘sensitivity paradox’ suggests three possible mechanisms:

First, the heightened involvement hypothesis: Employees who craft their jobs to increase autonomy and skill variety become more psychologically invested, leading to heightened awareness of all job aspects, including demands. This aligns with engagement theory, where deeper role involvement increases both positive and negative work experiences (Kahn, 1990; Schaufeli et al., 2002).

Second, the resource investment trap: COR theory typically assumes resource investment yields positive returns (Hobfoll, 1989, 2002). However, our findings suggest that investing resources in structural crafting may deplete cognitive resources needed for stress buffering, creating a net negative effect.

Third, the empowerment burden: Increased autonomy from structural crafting may paradoxically increase felt accountability for work outcomes, making objective demands feel more personally relevant and stressful (Cheong et al., 2016).

This finding aligns with the emerging literature on the potential dark side of job crafting (Harju et al., 2021) and suggests that empowerment through structural crafting may come with the cost of increased vigilance to work demands.

This unexpected finding suggests important boundary conditions for job crafting theory. While job crafting is generally positioned as beneficial, our results indicate that certain forms may increase rather than decrease stress perception. This calls for more nuanced theories that consider when and how job crafting might backfire.

The non-significant moderating effects of increasing challenging job demands (H2c, H3c) deserve careful consideration. Rather than dismissing these as simply ‘null findings,’ they may indicate boundary conditions for job crafting effects. Challenge-seeking may be orthogonal to stress perception—neither increasing nor decreasing sensitivity to objective conditions. This suggests that not all forms of job crafting operate through the same psychological mechanisms, supporting calls for more differentiated job crafting theories (Zhang & Parker, 2019).

Our findings expose tensions within established theories. COR theory assumes resource investment yields gains, yet structural crafting results suggest investment can increase stress sensitivity. JD-R positions job crafting as uniformly beneficial, yet we find differential and sometimes counterproductive effects.

The Stressor–Emotion Model (Spector & Fox, 2005) posits that perceived control moderates stress appraisal. Our findings suggest a more complex relationship: structural job crafting may increase objective control while simultaneously increasing subjective vulnerability to stressors. This apparent paradox can be resolved by recognizing that control and sensitivity are not opposites but potentially independent dimensions. Having more control over one’s work (through crafting) may make one more attuned to the demands that control must manage.

These tensions do not invalidate theories but suggest refinement needs—perhaps incorporating concepts like ‘optimal crafting levels’ or ‘crafting-context fit.’ Our study’s core contribution lies in demonstrating that the translation from objective work conditions to perceived stress is not uniformly moderated by job crafting. Rather than positioning job crafting as a simple intervention tool, our findings reveal it as a complex phenomenon that can both buffer and amplify stress perception depending on the specific crafting dimension and the aspect of work being evaluated (effort vs. reward). This nuanced view advances beyond simple main-effect perspectives of job crafting.

Organizations should recognize that job crafting interventions require nuanced implementation (Oprea et al., 2019; Van Wingerden et al., 2017). Social job crafting appears most consistently beneficial, both buffering effort perception and enhancing reward perception. However, structural crafting may have unintended consequences, particularly for experienced employees.

Rather than promoting job crafting universally, organizations should emphasize social crafting through collaborative cultures and feedback systems, monitor potential negative effects of structural crafting, provide support for employees engaging in autonomous job redesign, and recognize that different crafting strategies suit different contexts.

We must emphasize that our cross-sectional design precludes causal inferences. When we discuss job crafting ‘influencing’ or ‘moderating’ stress perception, these are theoretical propositions based on our conceptual model, not demonstrated causal effects. The relationships could be reciprocal (stress perception influencing crafting behaviors) or driven by unmeasured third variables. Longitudinal or experimental designs are essential to establish temporal precedence and causality.

Our sample of established Italian employees may limit generalizability to other cultural contexts or career stages. The focus on administrative positions excludes manual workers or executives who may craft differently.

While we controlled for demographic variables that showed significant associations with our key constructs, the substantial demographic differences observed warrant caution in generalization. The gender imbalance in our sample, combined with systematic differences in how men and women report work conditions and engage in job crafting, suggests that future research should examine whether the moderation effects we observed operate similarly across gender groups. Similarly, the age and education effects on job crafting behaviors indicate that career stage and human capital may influence not only the level but potentially the effectiveness of different crafting strategies.

Future research should examine the temporal dynamics of job crafting effects, investigate cultural and occupational boundary conditions, explore optimal combinations of crafting strategies, and test interventions that maximize benefits while minimizing the sensitivity paradox.

## 6. Conclusions

This study advances understanding of job crafting’s complex role in stress processes. Rather than uniformly beneficial, job crafting dimensions show differential effects on how objective work conditions translate into subjective experiences. The sensitivity paradox of structural crafting reveals that empowerment through job redesign may heighten stress awareness, while social crafting consistently provides stress-buffering benefits.

Our integrated ERI-JD-R framework demonstrates value in examining both content (what creates stress) and process (how individuals manage it). This dual-level approach reveals nuances missed by single-framework studies.

For practice, the distinction between factual job characteristics and their perception has important practical implications. Organizational interventions typically target factual characteristics (e.g., reducing overtime, clarifying roles), while individual interventions like job crafting target perceptual processes. Our results suggest that both levels require attention, as job crafting moderates but does not eliminate the relationship between factual indicators and perceived stress. Also, our findings suggest careful implementation of job crafting interventions, recognizing that not all crafting strategies are equally beneficial. Social job crafting emerges as the most promising avenue for helping employees manage workplace stress while maintaining awareness of organizational realities.

## Figures and Tables

**Table 1:** Descriptive statistics and intercorrelations of study variables.

Variable	M	DS	FIE	FIR	PE	PR	JC_ IStJR	JC_ ISoJR
FIE	2.83	1.08	-					
FIR	2.41	0.93	−0.302 **	-				
PE	2.85	0.66	0.329 **	−0.128 *	-			
PR	2.36	0.55	−0.536 **	0.212 **	−0.398 **	-		
JC_IStJR	5.72	0.80	0.049	0.008	0.038	−0.037	-	
JC_ISoJR	2.89	1.17	−0.017	−0.304 **	−0.080	0.210 **	0.080	-
JC_ICJD	4.81	1.21	0.145 **	−0.173 **	0.266 **	−0.135 **	0.538 **	0.206 **

Note. N = 376. FIE = factual indicators of effort; FIR = factual indicators of reward; PE = perceived effort; PR = perceived reward; JC_IStJR = increasing structural job resources; JC_ISoJR = increasing social job resources; JC_ICJDJC = increasing challenging job demands. * *p* < 0.05; ** *p* < 0.01 (two-tailed).

**Table 2:** Regression analysis of perceived effort on factual indicators of effort and job crafting factors and their interaction.

Predictors	*b*	SE	*t*	*p*	95% CI	
					LL	UL
FIE → PE	−0.292	0.241	−1.206	0.229	−0.767	0.184
JC_IStJR → PE	−0.209	0.121	−1.734	0.084	−0.447	0.028
FIE × JC_IStJR → PE	0.082	0.041	2.012	0.045	0.002	0.163
*R* ^2^	0.110 *					
*F*	6.241					
FIE → PE	0.342	0.070	4.886	0.000	0.204	0.480
JC_ISoJR → PE	0.093	0.071	1.315	0.189	−0.046	0.232
FIE × JC_ISoJR → PE	−0.046	0.020	−2.234	0.026	−0.086	−0.006
*R* ^2^	0.125 *					
*F*	8.228					
FIE → PE	0.145	0.141	1.030	0.304	−0.132	0.422
JC_ICJD → PE	0.123	0.086	1.424	0.155	−0.047	0.293
FIE × JC_ICJD → PE	0.005	0.027	0.170	0.865	−0.049	0.058
*R* ^2^	0.167 *					
*F*	12.050					

Note. N = 376. CI = confidence interval; LL = lower level; UL = upper level; FIE = factual indicators of effort; PE = perceived effort; JC_IStJR = increasing structural job resources; JC_ISoJR = increasing social job resources; JC_ICJDJC = increasing challenging job demands. Covariates (age, gender, education, and tenure) are omitted for parsimony. * *p* < 0.0001 (two-tailed).

**Table 3:** Regression analysis of perceived reward on factual indicators of reward and job crafting factors and their interaction.

Predictors	*b*	SE	*t*	*p*	95% CI	
					LL	UL
FIR → PR	−0.003	0.197	−0.016	0.987	−0.391	0.385
JC_IStJR → PR	−0.059	0.079	−0.745	0.457	−0.215	0.097
FIR × JC_IStJR → PR	0.016	0.036	0.460	0.646	−0.053	0.086
*R* ^2^	0.139 *					
*F*	6.418					
FIR → PR	−0.126	0.066	−1.922	0.055	−0.255	0.003
JC_ISoJR → PR	−0.091	0.060	−1.514	0.131	−0.200	0.027
FIR × JC_ISoJR → PR	0.091	0.021	4.433	0.000	0.051	0.132
*R* ^2^	0.232 *					
*F*	14.810					
FIR → PR	0.228	0.101	2.263	0.024	0.030	0.426
JC_ICJD → PR	0.030	0.053	0.570	0.569	−0.074	0.135
FIR × JC_ICJD → PR	−0.033	0.021	−1.600	0.111	−0.073	0.008
*R* ^2^	0.153 *					
*F*	7.097					

Note. N = 376. CI = confidence interval; LL = lower level; UL = upper level; FIR = factual indicators of reward; PR = perceived reward; JC_IStJR = increasing structural job resources; JC_ISoJR = increasing social job resources; JC_ICJDJC = increasing challenging job demands. Covariates (age, gender, education, and tenure) are omitted for parsimony. * *p* < 0.0001 (two-tailed).

## Data Availability

The data presented in this study are available upon reasonable request from the corresponding author.

## Abbreviations

The following abbreviations are used in this manuscript:

COR	Conservation of Resources
ERI	Effort–Reward Imbalance
JD-R	Job Demands–Resources
